# Biomarkers of Spinal and Bulbar Muscle Atrophy (SBMA): A Comprehensive Review

**DOI:** 10.3389/fneur.2018.00844

**Published:** 2018-10-10

**Authors:** Giorgia Querin, Peter Bede, Veronique Marchand-Pauvert, Pierre-Francois Pradat

**Affiliations:** ^1^Laboratoire d'Imagerie Biomédicale, CNRS, INSERM, Sorbonne Université, Paris, France; ^2^APHP, Département de Neurologie, Centre Référent SLA, Hôpital Pitié-Salpêtrière, Paris, France; ^3^Computational Neuroimaging Group, Academic Unit of Neurology, Trinity College Dublin, Dublin, Ireland; ^4^Northern Ireland Centre for Stratified Medicine, Biomedical Sciences Research Institute Ulster University, C-TRIC, Altnagelvin Hospital, Londonderry, United Kingdom

**Keywords:** SBMA, biomarkers, clinical trials, multisystem involvement, outcome measures

## Abstract

Spinal and bulbar muscular atrophy (SBMA), also known as Kennedy's disease, is a rare, X-linked, late onset neuromuscular disorder. The disease is caused by a CAG trinucleotide repeat expansion in the first exon of the androgen receptor gene. It is characterized by slowly progressive lower motor neurons degeneration, primary myopathy and widespread multisystem involvement. Respiratory involvement is rare, and the condition is associated with a normal life expectancy. Despite a plethora of therapeutic studies in mouse models, no effective disease-modifying therapy has been licensed for clinical use to date. The development of sensitive monitoring markers for the particularly slowly progressing pathology of SBMA is urgently required to aid future clinical trials. A small number of outcome measures have been proposed recently, including promising biochemical markers, which show correlation with clinical disability and disease-stage and progression. Nevertheless, a paucity of SBMA-specific biomarker studies persists, delaying the development of monitoring markers for pharmaceutical trials. Collaborative efforts through international consortia and multicenter registries are likely to contribute to the characterization of the natural history of the condition, the establishment of disease-specific biomarker panels and ultimately contribute to the development of disease-modifying drugs.

## Introduction

Spinal and bulbar muscular atrophy (SBMA), also known as Kennedy's disease, is a rare, X-linked, adult onset, neuromuscular disorder (1) characterized by slowly progressive lower motor neuron (LMN) degeneration, skeletal muscle pathology and by a spectrum of multi-organ involvement (2–4). The disease is caused by a CAG repeat expansion in the first exon of the androgen receptor (AR) gene encoding for a poly-glutamine (polyQ) tract. A repeat number higher than 38 is considered pathogenic (5). PolyQ-AR toxicity is hormone-dependent and CAG repeat size inversely correlates with age of symptom onset but not with disease progression rates (6, 7). Heterozygous female carriers of the mutation only present subtle signs of neuromuscular involvement such as muscle cramps and hand tremor (8, 9). The disease is rare, with an estimated prevalence of 3.5/100,000 male inhabitants in southern Europe (10, 11) but the presence of a founder effect is retained to cause considerable differences in the distribution of the disease in various geographical regions (12, 13). Subjects with minimal symptoms and the relatively limited awareness of the condition make it likely that the real prevalence of SBMA is underestimated.

Despite several promising therapeutic studies (14), no disease-modifying treatment currently exists for SBMA. Similarly to SMA, the lack of sensitive monitoring markers for the slow progression rates of SBMA is one of main the barriers to successful clinical trials (15, 16). The objective of this work is the systematic review of candidate biomarkers in SBMA and the appraisal of their potential in clinical management and pharmaceutical trials.

## The neurological presentation

Limb weakness is present in 97% of SBMA cases. It usually appears at the of age of 35–40 and starts typically proximally in the lower limbs (2, 3, 6, 17). However, tremors, muscle cramps, myalgia, gynecomastia, and exercise intolerance are often reported long before the onset of frank limb weakness (17, 18). Clinical signs of LMN involvement, such as fasciculations, muscle cramps, and atrophy are invariably present. Proximal muscles are predominantly affected, leading to difficulties in climbing stairs and getting up from a sitting position. Motor impairment is usually slowly progressive (19) and survival is only slightly reduced (6, 17). In addition to limb muscle wasting, fasciculations, and decreased deep tendon reflexes, clinical features often include a high-frequency postural hand tremor and postural leg tremor (20).

Bulbar impairment occurs in about 10–30% of patients at the onset of the disease (17), but it is present in the majority of the patients at later stages. It slowly progresses over time and may lead to aspiration pneumonia, which is a frequent cause of death in SBMA (6). Dysphagia is due to impaired oro-pharyngeal phase of deglutition (21), and is associated with tongue's muscles weakness, fasciculations, and atrophy (21). Dysarthria is characterized by hypernasality secondary to incomplete soft palate elevation and is associated with dysphonia. Speech impairment can evolve into markedly reduced intelligibility. Facial weakness and asymmetry, perioral fasciculations, myokymia, and jaw drop are also common clinical features (21–23). Recurrent laryngospasms have been noted in up to 47% of SBMA patients (24).

The presence of a distal sensory neuropathy is a hallmark feature of the disease (25) which has been described in post-mortem studies (26), sural nerve biopsies (27), and neurophysiology (28). The sensory neuropathy may be asymptomatic or manifests in distal numbness and paraesthesia in the lower limbs and reduced sensation for vibration. Neurophysiological examination readily detects reduced or absent sensory action potentials (SAPs) (28, 29). Degeneration of small myelinated and unmyelinated fibers may explain the high incidence of neuropathic pain (30) in SBMA.

## Multisystem involvement

Complex multi-organ involvement is a hallmark feature of SBMA. The core non-neurological features of SBMA include gynecomastia, testicular atrophy, reduced fertility and erectile dysfunction. Dysfunction of the AR protein leads to partial androgen insensitivity (31), manifesting in erectile dysfunction (3), gynecomastia and reduced fertility (31, 32). Testosterone and dehydro-epiandrosterone sulfate (DHEAS) are elevated in up to 38% of patients (32). The Androgen Sensitivity Index (ASI) (LH × testosterone), which reflects androgen resistance, is found to be increased in almost half of the patients (3, 32). DHEAS is thought to correlate with CAG repeat number as well as disease duration (32). Metabolic syndrome with increased BMI, elevated serum cholesterol, triglycerides, and fasting glucose is also a key feature of the disease (3, 31–33) and insulin resistance is associated with disease severity (34). Liver involvement with steatosis and sometimes inflammation has been described (33), but the risk of progression to liver fibrosis is unclear. Recurrent urinary symptoms and incomplete bladder emptying may affect more than the third of male SBMA patients even in the absence of benign prostatic hyperplasia, which is likely to be explained by pelvic floor and bulbuocanvernosus muscle dysfunction (3). While there is no evidence of a primary cardiomyopathy in SBMA (35), Brugada-like ECG abnormalities have been reported in almost half of the patients in a large Japanese cohort (36). Obstructive sleep apnea (OSA), poor sleep quality and periodic limb movements in sleep have also been reported (37).

## Biomarkers in SBMA

A biomarker is a parameter that can be measured accurately and reproducibly and used as an indicator of normal biological processes, pathogenic processes, or pharmacologic responses to a therapeutic intervention (WHO definition, 1998). An ideal biomarker should have a predictive value and capture subtle changes over relatively short periods of time. Additional requirements to biomarkers include cost-effectiveness, non-invasiveness, and reproducibility (38, 39). It is generally agreed that no single biomarker is suitable for diagnostic, prognostic and monitoring roles and a panel of several markers may be better suited as multirole indicators (40). SBMA is a rare and slowly progressing condition, therefore the development of sensitive outcome measures would enable smaller sample-size and shorter duration of pharmaceutical trials (41, 42).

## Biomarkers of neurological involvement in SBMA

In recent years, an unprecedented interest has developed in the standardized assessment of neuromuscular performance in SBMA, evaluation of novel therapeutic strategies (14) and in the launch of national SBMA registries (42, 43). Many of the commonly used instruments, such as the MRC score, respiratory function parameters, the modified Norris scale, ALSFRS-r, Quantitative Myasthenia Gravis Score etc. are non-specific to SBMA, yet remain widely utilized. As these tools have been developed for other conditions, new batteries of tests have been recently proposed to specifically appraise disability in SBMA (Table 1).

**Table 1:** Research studies considering motor and bulbar skills-related outcome measures.

**Primary outcome measure**	**Reference number**	**Authors**	**Other outcome measures in the study**	**Type of study**	**Number of patients**	**Duration of follow-up**
**MOTOR SKILLS-RELATED OUTCOME MEASURES**						
6MWT	(44)	Takeuci et al.	Modified Norris score, ALSFRS-R, grip strength	Observational, longitudinal study	35 at baseline, 24 at follow-up	12 months
	(45)	Querin et al.	MMT, ALSFRS-R, FVC	Pilot, unblinded pharmacological trial (Clenbuterol)	20	12 months
AMAT	(46)	Harris-Love et al.	QMA, 2MWT, ADL assessment, SF-36v2	Observational, cross-sectional study	55	/
	(47)	Shrader et al.	QMA, STS test, Timed up and Go test, Balance tests, SF-36v2, Beck depression scale, serum CK, IGF-1 and testosterone	Randomized, evaluator-blinded pharmacological trial (Physical exercise)	50	12 weeks
SBMAFRS	(48)	Hashizume et al.	ALSFRS-R, Modified Norris Score	Observational, longitudinal study	80	12 months
	(49)	Querin et al.	MMT, 6MWT, ALSFRS-R	Observational, longitudinal study	60	8 weeks
1234 scale	(50)	Lu et al.	ALSFRS-R	Observational, longitudinal study	81	32 months
ALSFRS-R	(51)	Banno et al.	VF, MMT, FVC, serum CK, AST, ALT, Beck depression scale, 1C2-positive cells in scrotal skin biopsies	Randomized, double-blinded pharmacological trial (Leuprorelin)	50	48+96 weeks
QMA	(52)	Fernández-Rhodes et al.	AMAT, MMT, 2MWT, SF-36v2, IIEF, MUNE, CMAP VF, FVC, serum CK and testosterone	Randomized, double-blinded pharmacological trial (Dutasteride)	50	24 months
Hand grip strength	(53)	Hijikata et al.	Modified QMG score, ALSFRS-R, SBMAFRS, 15-foot timed-walk test, rise-from-bed test, swallowing questionnaires, FVC, Multidimensional Fatigue Inventory, urinary 8-OHdG	Randomized, double-blinded pharmacological trial (Creatine Monohydrate)	45	8 weeks
**BULBAR FUNCTION-RELATED OUTCOME MEASURES**						
Tongue pressure	(54)	Mano et al.	Modified Norris score, ALSFRS-R, QMA, grip strength, MMT, modified QMG score, VF, swallowing questionnaires, timed walk test	Observational, cross-sectional study (validity of tongue pressure as marker of dysphagia)	47	/
	(55)	Mano et al.	VF, modified QMG score, ALSFRS-R, serum CK and testosterone	Non-randomized, interventional study (head lift exercises)	6	12 weeks
VF	(56)	Hashizume et al.	ALSFRS-R, SBMAFRS, swallowing questionnaires, Limbs Norris score, Bulbar Norris score	Observational, longitudinal study	111	30 days
	(57)	Katsuno et al.	ALSFRS-R, 6MWT, modified QMG score, 1C2-positive cells in scrotal skin biopsies, serum CK and testosterone, ALSAQ-5 score	Randomized, double-blinded pharmacological trial (Leuprorelin)	204	12 months
FEES	(21)	Warnecke et al.	MMT, modified Rankin scale	Observational, cross-sectional study	10	/
**INSTRUMENTAL OUTCOME MEASURES**						
MUNE	(58)	Suzuki et al.	Limbs Norris score, Bulbar Norris score, ALSFRS-R, grip strength	Observational, longitudinal study	52	12 months
	(59)	Lehky et al.	CMAP, SMUP	Observational, cross-sectional study	54	/
CMAP and SNAPs	(29)	Suzuki et al.	Limbs Norris score, Bulbar Norris score, ALSFRS-R, spinal cord tissue specimens	Observational, cross-sectional study	106	/
Muscle MRI	(60)	Hamano et al.	/	Observational, cross-sectional study	3	/

*ALSFRS-R, Amyotrophic Lateral Sclerosis functional rating scale-revised; MMT, manual muscle testing; FVC, forced vital capacity; QMA, quantitative muscle assessment, 2 or 6MWT, 2 or 6 minutes-walk-test; ADL, activity of daily living; DXA, Dual-energy X-ray absorptiometry, urinary 8-OHdG, 8-hydroxydeoxyguanosine; VF, videofluoroscopy; AMAT, adult myopathy assessment tool; IIEF, International Index of erectile function; MUNE, motor unit number estimate; CMAP, compound motor action potential; CK, creatine-kinase; QMC score, quantitative myasthenia gravis score; SMUP, single motor unit potential*.

### 6-minute-walk-test (6MWT)

The 6-minute-walk-test (6MWT) was proposed as an accurate marker of disease progression (44). It measures the distance a person can walk within 6 min and is regarded as a composite proxy of cardiopulmonary and neuromuscular abilities (61). Due to its relative simplicity and cost-effectiveness it has been widely adopted as an outcome measure in several neuromuscular conditions, such SMA and myopathies (62, 63). The 6MWT is traditionally considered the most reliable marker of motor impairment in SBMA, it reliably captures a 10% decline over 1 year (44) and has been used as a primary outcome measure in clinical trials (45, 57). A shorter version of the test, the “2-MWT,” also exists and is thought to be reliable (63).

### Adult myopathy assessment tool (AMAT)

The Adult myopathy assessment tool (AMAT) is a performance-based instrument composed of functional and endurance subscales (46). AMAT provides a comprehensive evaluation of motor function, and muscle fatigue, which is a key facet of disability in SBMA (64). One of the strengths of AMAT is that it can also be applied to non-ambulatory patients. It is widely used in both SBMA registers (43) and in clinical trials (47, 52).

### Sbma functional rating scale (SBMAFRS)

The SBMA functional rating scale (SBMAFRS) SBMAFRS is a recently validated scale (48, 49), which has been developed from the ALSFRS-r (65) and specifically adapted for the disability profile of SBMA. It is a questionnaire-based scale that measures physical function in activities of daily living (ADL) and consists of five main domains measuring bulbar, upper-limb, lower-limb, truncal, and respiratory function. The SBMAFRS has proven to be more sensitive than the ALSFRS-r in evaluating SBMA patients with moderate motor deficits (48).

### 1234-scale

The 1234-scale is another questionnaire-based scale based on the ALSFRS-r, which focuses on SBMA-associated motor disability (50). It includes items such as the ability to do push-ups, ability to run and to stand up from a squatting position. The 1234-scale has shown good internal validity and high reliability (50), but its sensitivity as a monitoring marker has not been confirmed.

### Quantitative muscle strength assessment (QMA)

Manual muscle testing (MMT) is commonly used to describe muscle weakness in neuromuscular conditions even though it is highly evaluator-dependent (66). A number of more objective techniques are available to evaluate muscle strength quantitatively in the four limbs (67). Grip strength measured by a handheld dynamometer is one of the simplest and most reproducible QMA parameters. Significant changes in grip strength have been observed in a 3-year longitudinal study of SBMA (19), but progressive changes have not been captured over a 1-year follow-up (44). QMA of maximal voluntary isometric muscle strength has been repeatedly proposed as an outcome measure for clinical trials (46, 47, 52, 54), but its efficacy as a biomarker is limited by considerable inter-centers variability.

### Videofluoroscopy (VF)

Videofluoroscopy (VF) is routinely used to evaluate dysphagia in a range of neurological conditions. In SBMA, VF can reliably detect the impairment of the oral phase of deglutition confirming large amount of oral barium residue (56). VF has been previously used in clinical trials (51, 55, 68), but the lack of standardization makes it less suitable for robust multicenter studies.

### Fiber endoscopic evaluation of swallowing

Fiber endoscopic evaluation of swallowing has also been assessed as a candidate biomarker of bulbar impairment, but the diagnostic and prognostic value of the technique is yet to be validated (21).

### Tongue pressure

Tongue pressure measurements using an electronic device has been proposed as a biomarker of dysphagia in SBMA, and has been shown to be a low-cost and reliable way of detecting tongue weakness early in the course of the disease (54). An important limitation is that it is susceptible to a ceiling effect in subjects with severe bulbar impairment. Nevertheless, it has been used successfully in a trial of head-lift exercises as a possible rehabilitation strategy in SBMA-associated dysphagia (55).

### Electrophysiology

Standard electrophysiology measures are routinely used in the diagnostic work-up of SBMA, but they exhibit limited sensitivity to longitudinal changes (28). This is somewhat unexpected given the correlation between CAG repeat numbers and electrophysiological parameters (29). Quantitative Motor Unit Number Estimation (MUNE) techniques have emerged as a promising way of quantifying motor neuron loss in a number of motor neuron diseases (69, 70). Significant MUNE reductions have been shown in SBMA patients both in cross-sectional and longitudinal study designs, making it one of the most promising candidate outcome measures (58, 59). MUNIX is a more recent, non-invasive method of quantifying motor neuron loss, that has already been utilized in ALS (71), peripheral neuropathies (72), and more recently in adult SMA patients (16). The motor unit size index (MUSIX) (CMAP amplitude/MUNIX) is increasingly accepted as a measure of compensatory collateral sprouting. This technique has not been tested in SBMA yet, but is likely be a promising tool in the evaluation of longitudinal motor neurons loss.

### Quantitative muscle MRI

While quantitative muscle MRI would be an obvious candidate marker of disease progression in SBMA, there is a surprising scarcity of such studies. Existing studies have shown that muscle imaging can effectively detect muscle pathology in distal leg muscles which is less obvious on clinical assessment (60).

### Spinal cord imaging

Spinal cord imaging has seen unprecedented advances in recent years and has been applied successfully to other motor neuron diseases such as ALS (73–75), and SMA (15) to characterize gray (76) and white matter pathology (77). There is an ongoing study to test its efficacy in SBMA patients (NCT02885870).

### Quantitative brain imaging

Quantitative brain imaging studies demonstrated white matter alterations in the corticospinal tracts (CST), limbic system (78, 79), brainstem and cerebellum (80). Voxel-based morphometry (VBM) of SBMA cohorts revealed gray matter atrophy in the frontal lobes and in the brainstem (78–81). Frontal hypometabolism has been detected by positron-emission-tomography (PET) (82). These studies confirm the multisystem nature of SBMA-associated pathology, and that neurodegeneration is not limited to LMNs but involve the CSTs and widespread cerebral regions. Despite imaging evidence of extra-motor involvement, neuropsychological studies have only detected subtle frontal dysfunction in small study populations (83, 84) which were not confirmed in larger cohorts (85, 86).

## Biomarkers of multisystem involvement in SBMA

### Increased serum CK levels

Increased serum CK levels have been reported by almost every SBMA study and support the hypothesis of a primary myopathy in SBMA (87, 88). Elevated serum CK levels can be detected prior to symptom onset (89) and may be most marked around disease manifestation (18, 19). Nevertheless, no correlation was found between serum CK levels and age of onset, CAG repeat numbers, disease duration or rate of progression (6, 19). As a result, CK levels are thought to be useful as part of the diagnostic workup, but of limited use in monitoring disease progression.

### Transaminases levels

Transaminases levels have also consistently been shown to be raised in SBMA including the pre-symptomatic phase of the disease (89), but they do not correlate with the progression of the neurological symptoms. The clinical significance of raised transaminases in SBMA is a topic of debate and its prognostic value remains to be established (33).

### Serum creatinine level

Serum creatinine level has also been proposed as a potential biomarker (90) despite its lack of specificity to SBMA. It tends to be reduced in the pre-symptomatic and symptomatic phases of the disease (91) and correlate well with parameters of motor impairment (6, 19, 91).

### Proxies of metabolic syndrome and insulin resistance

Proxies of metabolic syndrome and insulin resistance are considered closely associated with primary molecular disease mechanisms. The homeostasis model assessment of insulin resistance (HOMA-IR) index correlated significantly with motor function parameters in one study (34), but this relationship has not been confirmed by others (32). **Hormones levels** and **ASI** (Androgen Sensitivity Index) have also been repeatedly proposed as markers of SBMA. Free testosterone levels correlate with muscle strength in one study (2) but it does not correlate with CAG repeat numbers or disease progression according to others (57). DHEAS levels have been linked to disease duration (91).

### Skin biopsies

Skin biopsies have been performed in some clinical trials to evaluate changes in the frequency of anti-polyQ antibody-positive cells after treatment (57). This index may be sensitive to changes during pharmacological treatment but the methodology is inherently invasive and poorly harmonized across different centers.

### Adipose tissue quantification

A recent study proposed adipose tissue quantification using whole-body MRI and reported significant subcutaneous fat accumulation in SBMA patients. This correlated both with CAG repeat lengths, disease duration and progression rates (32). These data suggest that adipose tissue MRI may be an additional marker of multisystem involvement in SBMA.

## Discussion and future perspectives

**Figure 1:**
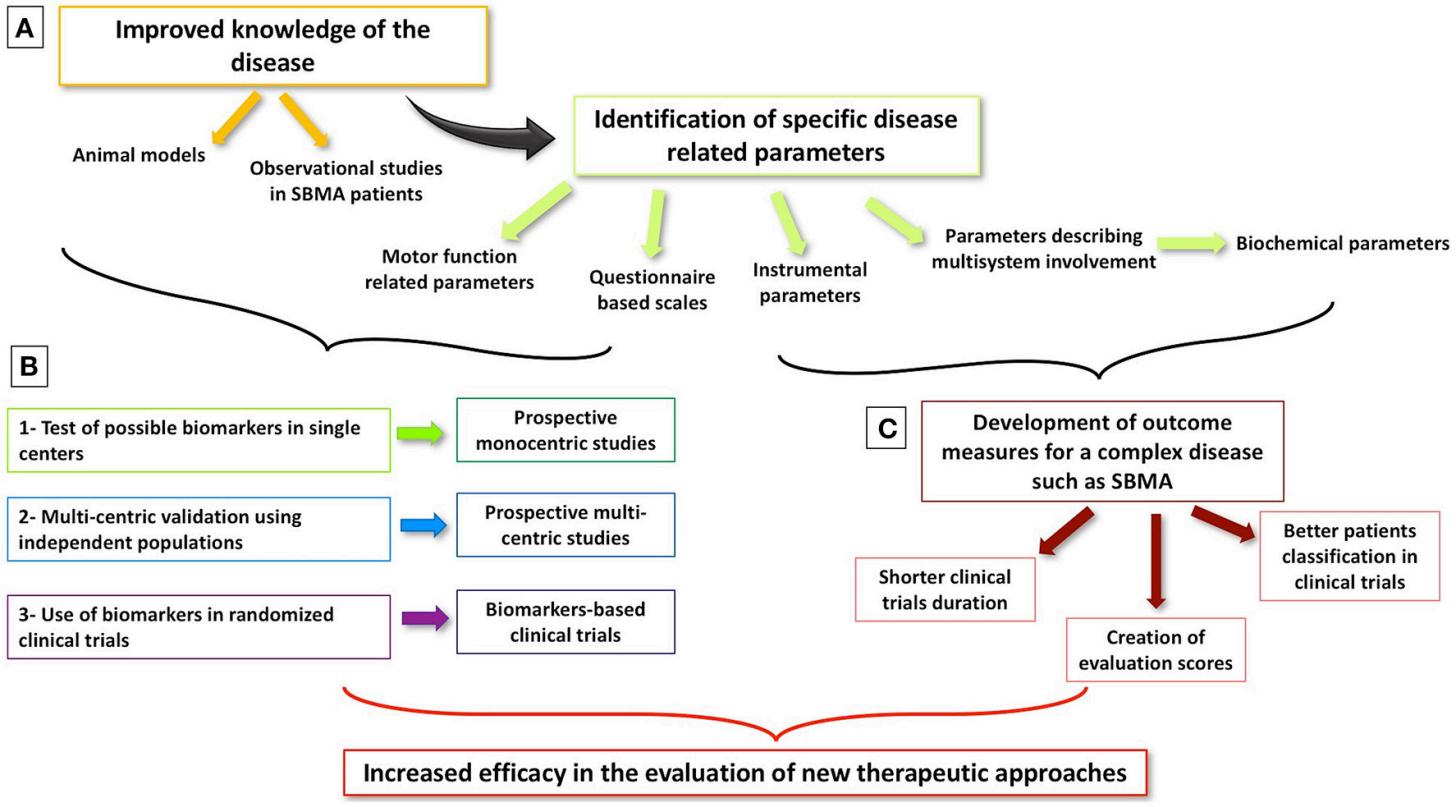
Milestones of biomarker development in SBMA. **(A)** Better knowledge of SBMA through animal models and observational studies allows the identification of possible biomarkers of disease status and of its progression. **(B)** Different steps are needed to develop and validate a biomarker in order to make it a reliable outcome measure in clinical trials. **(C)** Considered the complexity of SBMA and its multi-system presentation, the development of global biomarkers, including both motor function and biochemical parameters, is warranted with the aim of improving the efficacy of upcoming clinical trials.

Interest in SBMA biomarkers has grown steadily in recent years, fuelled both by accruing knowledge about pathogenesis and novel therapeutic strategies (14, 42). SBMA is now widely recognized as a multisystem syndrome (3). A multitude of studies focus on multi-organ involvement, and the systemic phenotype is now considered just as relevant as the neurological manifestations. It is increasingly recognized that non-neurological features of the disease have an equally important impact on the patients' quality of life (3, 31–34, 87, 88, 91, 92). Until now, clinical trials on SBMA focused almost exclusively on the treatment of motor symptoms (14, 45, 47, 51–53, 55, 57, 68, 92, 93), but a shift to targeted molecular therapies (94) and focus on systemic processes are likely to be witnessed in the near future. From a clinical trial perspective, ideal biomarkers should undergo robust validation, sensitivity and specificity profiling, and sampling and measurement harmonization across different centers. Crucially, candidate markers should be able to detect the subtle changes expected after the administration of a specific treatment (95). Given the particularly slow progression rates observed in SBMA, the definition of an effective outcome measures is challenging. The integration of neurological, metabolic, and endocrine indicators seems essential into composite biomarker panels in addition to functional scales. Serum creatinine levels appear to correlate strongly with motor impairment and HOMA-IR index with disease duration (34). The convincing validation of these parameters and their use as effective outcome measures in clinical trials will require robust multicenter study designs (96) (Figure 1).

Furthermore, the comparison of the specificity profile of candidate biomarkers seems essential to define their roles in clinical applications. The establishment of national and international SBMA registers is a clear priority which will be an invaluable resource for future SBMA research (42). As in other neurodegenerative conditions (95, 96), the integration of clinical, molecular, imaging and neurophysiological markers may be required for assessing the efficacy of disease-modifying interventions (95, 96). To conclude, we underline the relevance of considering both motor (muscle force evaluation, questionnaire based scales, and performed tasks) and biochemical parameters as possible outcome measures for a multi-system and complex pathology as SBMA. Beyond their monitoring roles, validated biomarkers will also aid patient stratification upon entry into pharmacological trials (97).

## Author contributions

The paper was drafted by GQ and PB and has been reviewed for intellectual content by VM-P and P-FP.

### Conflict of interest statement

The authors declare that the research was conducted in the absence of any commercial or financial relationships that could be construed as a potential conflict of interest.
